# Enterovirus-71 Virus-Like Particles Induce the Activation and Maturation of Human Monocyte-Derived Dendritic Cells through TLR4 Signaling

**DOI:** 10.1371/journal.pone.0111496

**Published:** 2014-10-31

**Authors:** Yu-Li Lin, Yu-Chen Hu, Cheng-Chao Liang, Shih-Yeh Lin, Yu-Chih Liang, Hui-Ping Yuan, Bor-Luen Chiang

**Affiliations:** 1 Department of Medical Research, National Taiwan University Hospital, Taipei, Taiwan; 2 Department of Chemical Engineering, National Tsing Hua University, Hsinchu, Taiwan; 3 Department of Internal Medicine, Far Eastern Memorial Hospital, Taipei, Taiwan; 4 School of Medical Laboratory Science and Biotechnology, College of Medical Science and Technology, Taipei Medical University, Taipei, Taiwan; 5 Graduate Institute of Clinical Medicine, College of Medicine, National Taiwan University, Taipei, Taiwan; University of California, San Francisco, United States of America

## Abstract

Enterovirus 71 (EV71) causes seasonal epidemics of hand-foot-and-mouth disease and has a high mortality rate among young children. We recently demonstrated potent induction of the humoral and cell-mediated immune response in monkeys immunized with EV71 virus-like particles (VLPs), with a morphology resembling that of infectious EV71 virions but not containing a viral genome, which could potentially be safe as a vaccine for EV71. To elucidate the mechanisms through which EV71 VLPs induce cell-mediated immunity, we studied the immunomodulatory effects of EV71 VLPs on human monocyte-derived dendritic cells (DCs), which bind to and incorporate EV71 VLPs. DC treatment with EV71 VLPs enhanced the expression of CD80, CD86, CD83, CD40, CD54, and HLA-DR on the cell surface; increased the production of interleukin (IL)-12 p40, IL-12 p70, and IL-10 by DCs; and suppressed the capacity of DCs for endocytosis. Treatment with EV71 VLPs also enhanced the ability of DCs to stimulate naïve T cells and induced secretion of interferon (IFN)-γ by T cells and Th1 cell responses. Neutralization with antibodies against Toll-like receptor (TLR) 4 suppressed the capacity of EV71 VLPs to induce the production of IL-12 p40, IL-12 p70, and IL-10 by DCs and inhibited EV71 VLPs binding to DCs. Our study findings clarified the important role for TLR4 signaling in DCs in response to EV71 VLPs and showed that EV71 VLPs induced inhibitor of kappaB alpha (IκBα) degradation and nuclear factor of kappaB (NF-κB) activation.

## Introduction

Enterovirus 71 (EV71) is responsible for seasonal epidemics of hand-foot-and-mouth disease and is associated with a high mortality rate [1], aseptic meningitis, and severe neurological complications [2] in young children. Outbreaks of EV71 infection in Taiwan, Japan, and Singapore have killed more than 100 children over the past decade [3]. However, no antiviral drugs or vaccines effective against EV71 are available, and measures to prevent EV71 epidemics rely exclusively on public surveillance. Immunization with inactivated intact EV71 virus (10 µg/mouse) induces immune responses and confers protection against lethal EV71 infection in mouse models [4]. However, the use of a live attenuated vaccine in humans raises safety concerns regarding possible adverse effects, especially in immunocompromised individuals. Vaccines based on recombinant DNA technology promise to mitigate these risks. However, DNA vaccines (100 µg/mouse) and recombinant protein vaccines (10 µg/mouse) based on VP1, the most potent antigen (Ag) on the EV71 virus, induce poorer immune responses than inactivated virus vaccines and fail to effectively protect mice against infection by EV71 [4].

EV71 is a nonenveloped, single-stranded RNA virus within the *Picornaviridae* family. The enterovirus genome contains P1, P2, and P3 regions. The P2 and P3 regions encode nonstructural proteins (e.g., 3CD), which are responsible for virus replication and virulence, whereas the P1 region encodes the P1 precursor, which can be cleaved by the 3CD protease into VP0, VP1, and VP3. These three proteins spontaneously assemble into an icosahedral procapsid that packs the RNA genome into the provirion [5].

Virus-like particles (VLPs) are particles that comprise viral capsid proteins but are devoid of viral nucleic acids. The absence of nucleic acids mitigates the potential side effects associated with immunization with an attenuated virus. The repetitive, high-density display of viral Ags and epitopes on the surfaces of VLPs usually elicits strong immune responses similar to those triggered by authentic viruses [6], [7]. To develop EV71 vaccines and VLPs as a potential vaccine platform [8], we previously constructed a recombinant baculovirus (Bac-P1-3CD) that co-expresses the P1 and 3CD proteins of EV71, and we showed that infection of insect cells with this virus leads to the cleavage of P1 by the 3CD protease into individual proteins (VP0, VP1, and VP3) and the self-assembly of EV71 VLPs within the cells [9], [10]. After purification by ultracentrifugation, the dispersed EV71 VLPs are indistinguishable from the authentic virus in size, appearance, composition, and surface epitopes, as determined by sodium dodecyl sulfate-polyacrylamide gel electrophoresis (SDS-PAGE), western blot analysis, transmission electron microscopy, and immunogold labeling [10].

Dendritic cells (DCs) belong to a major class of professional Ag-presenting cells; their primary function is to capture, process, and present Ags to unprimed T cells [11]. Immature DCs reside in nonlymphoid tissues where they can capture and process Ags. Thereafter, DCs migrate to the T-cell-containing areas of lymphoid organs where they lose their Ag-processing activity and mature to become potent immunostimulatory cells [12]. The maturation of DCs is critical for the induction of Ag-specific T-lymphocyte responses and may be essential for the development of human vaccines that rely on T-cell immunity. Fully mature DCs express high levels of MHC class II and costimulatory molecules (CD40, CD80, and CD86) on their surfaces, but have less capacity to internalize Ags than immature DCs [12]. Mature DCs present increased levels of CD83, a specific marker for DC maturation [13]. Various stimuli, such as pro-inflammatory cytokines (e.g., tumor necrosis factor [TNF]-α and interleukin [IL]-1), the CD40 ligand, bacterial products (e.g., lipopolysaccharide [LPS] and unmethylated DNA with the CpG motif), and contact sensitizers, can induce DC maturation both *in vivo* and *in vitro* [14]–[16]. Thus, DCs function as outposts of immune surveillance in that they trigger primary immune reactions against infectious pathogens, including viruses.

Recently, we showed that monkeys intramuscularly immunized with EV71 VLP-based vaccines presented potent humoral and cellular immune responses to both EV71 VLPs and EV71 virions [17]. However, the mechanisms through which VLPs induce cell-mediated immunity remain unknown. To address this issue, the present study used immature human monocyte-derived DCs to assess the early events that occur after DCs encounter noninfectious EV71 VLPs and to investigate the ability of DCs to initiate primary T-cell responses. We found that EV71 VLPs trigger Toll-like receptor (TLR) 4 and nuclear factor kappaB (NF-κB) signaling to induce phenotypic and functional maturation of DCs, which induces Th1-dominated immune responses. We believe that our findings might help to better elucidate the function of DCs in the pathogenesis of EV71 and the potential utility of VLPs for vaccination against EV71.

## Materials and Methods

### Reagents

Escherichia coli LPS (L8274, Escherichia coli) was purchased from Sigma-Aldrich (St. Louis, MO, USA). Neutralization antibodies (Abs) against TLR2 (clone TL2.1) and TLR4 (clone HTA125) were purchased from eBiosciences (San Diego, CA, USA). To rule out the possibility of endotoxin contamination in the EV71 VLP samples, the chromogenic Limulus Amebocyte Lysate assay (GenScript, NJ, USA) was used to determine the LPS content. The level of endotoxin in the EV71 VLP sample was lower than 1.0 endotoxin unit/mL.

### Preparation of EV71 VLPs

EV71 VLPs prepared from Sf-9 or Hi-5 cells infected with recombinant baculovirus were purified via affinity chromatography as previously described [10].

### Generation of human DCs

DCs were generated from peripheral blood mononuclear cells (PBMCs) of healthy donors, as described previously [18]. This study was approved by the Research Ethics Committee of the National Taiwan University Hospital and informed consent was obtained from all subjects. The research process, including the collection and storage of blood and the isolation of immune cells was explained in detail to every participant. Every participant provided written informed consent to participate in this study.

Briefly, PBMCs were obtained by Ficoll-Hypaque density gradient centrifugation, and the low-density fraction from the 42.5%–50% interface was recovered. CD14^+^ cells were purified by positive selection using anti-CD14^+^ microbeads in conjunction with the AutoMACS system by following the manufacturer’s instructions (Miltenyi Biotec, Bergisch Gladbach, Germany). CD14^+^ cells were cultured at 1×10^6^ cells/mL in RPMI 1640 (containing 10% fetal bovine serum [FBS], 2**mM l-glutamine, 100**U/mL penicillin, 100**U/mL streptomycin, and 25**mM HEPES) in 24-well plates with granulocyte-macrophage colony-stimulating factor (GM-CSF, 20**ng/mL) and IL-4 (20**ng/mL; PeproTech, NJ, USA). Fresh medium containing GM-CSF and IL-4 was added every 2 or 3 days. Human monocyte-derived DCs were routinely used on the fifth day of culture for treatment with EV71 VLPs.

### Binding of EV71 VLPs

**Figure 1:**
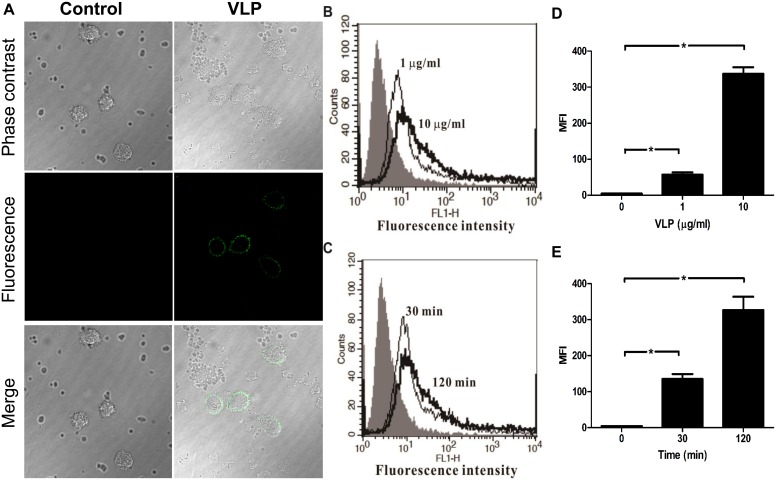
Binding of EV71 VLPs to immature human monocyte-derived DCs. For the analysis of concentration-dependent effects, human monocyte-derived DCs were incubated with 1 or 10**µg/mL EV71 VLPs for 30**min on ice to allow binding to the cell surface. For the analysis of time-dependent effects, DCs were incubated with 5**µg/mL of EV71 VLPs for 30 or 120**min on ice. Detection was facilitated using an anti-EV71 Ab and anti-mouse-IgG Ab (labeled with Alexa Fluor 488). Levels of fluorescence were determined using immunofluorescence confocal microscopy (A) and FACS analysis (B, C). (B) and (C) indicate the respective concentration-dependent and time-dependent effects of the binding of EV71 VLPs to immature human monocyte-derived DCs. The control cells (no VLPs; filled histogram) were stained with the same primary antibody (anti-EV71 Ab) followed by a secondary antibody. (D) and (E) indicate the bar diagram of mean fluorescence intensity (MFI) with statistics for concentration- and time-dependent binding of EV71 VLPs to DCs. Representative data from three different donors with similar results are shown. Values represent the means ± SEs of triplicate samples, and asterisks indicate statistically significant differences (*, *P*<0.05).

DCs were incubated with different concentrations of EV71 VLPs for different times (as indicated in the legend for Figure 1) on ice. After washing, an anti-EV71 Ab (Millipore, USA) was added, and the cells were incubated for 30**min at 4°C. Unbound anti-EV71 Ab was washed away, anti-mouse IgG Ab (labeled with Alexa Fluor 488; BD) was added, and the cells were incubated for 30**min at 4°C. Fluorescence was examined by flow cytometry and confocal microscopy (ZEISS LSM 510 META; Carl Zeiss Inc., Thornwood, NY, USA).

### Uptake of EV71 VLPs

**Figure 2:**
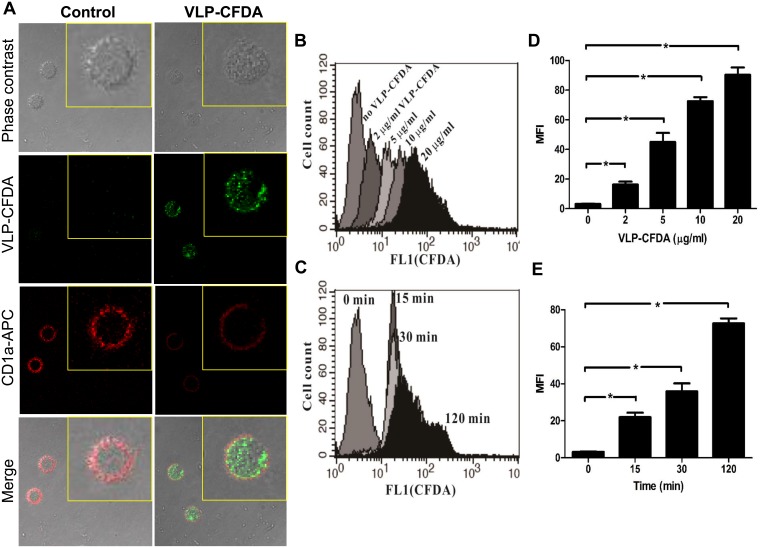
Uptake of EV71 VLPs by immature human monocyte-derived DCs. For the analysis of concentration-dependent effects, DCs were collected and incubated with different concentrations of CFDA-labeled EV71 VLPs (0, 2, 5, 10, or 20**µg/mL) for 120**min at 37°C. For the analysis of time-dependent effects, DCs were incubated with 10**µg/mL of CFDA-labeled EV71 VLPs for different time points (0, 15, 30, or 120**min) at 37°C. At the end of the incubation time, fractions were collected and fixed before double staining with an allophycocyanin-labeled anti-CD1a Ab. Fluorescence was examined by immunofluorescence confocal microscopy (an enlargement of a single cell is shown) (A) and FACS analysis (B, C). (B) and (C) indicate the respective concentration-dependent and time-dependent uptake of EV71 VLPs to immature human monocyte-derived DCs. (D) and (E) show bar diagrams of MFIs with statistics for concentration- and time-dependent uptake of EV71 VLPs. Representative data from three different donors with similar results are shown. Values represent the means ± SEs of triplicate samples, and asterisks indicate statistically significant differences (*, *P*<0.05).

EV71 VLPs were labeled with carboxyfluorescein diacetate (CFDA; Vybrant CFDA Cell Tracer Kit; Invitrogen, NY, USA) for 4**h at 25°C. Excess CFDA was removed by dialysis against 4**L of phosphate-buffered saline (PBS)/0.5**M NaCl overnight at 4°C. DCs were collected and incubated with various concentrations of EV71 VLPs-CFDA for 120**min at 37°C, or DCs were incubated with 10**µg/mL EV71 VLPs-CFDA for various time point (as indicated in the legend for Figure 2). After incubation, fractions were collected, fixed with paraformaldehyde, and then double stained with an allophycocyanin-labeled anti-CD1a Ab (BD Diagnostic, Sparks, MD, USA). Fluorescence was examined using flow cytometry and confocal microscopy (ZEISS LSM 510 META). To exclude remaining CFDA, DCs were incubated with PBS, which had been labeled with CFDA using a procedure similar to that described for EV71 VLPs, and excess CFDA was removed by dialysis. This sample was used as a control.

### Determination of cytokine levels

Levels of IL-12 p70, IL-12 p40, IL-10, IL-5, IFN-γ, and IL-8 in the supernatants from cultures of DCs, T cells, or HEK293 cells were assayed using an enzyme-linked immunosorbent assay (ELISA) kit (R&D, USA), according to the manufacturer’s instructions.

### Flow cytometry

DCs were harvested and washed with cold buffer (PBS containing 2% fetal calf serum and 0.1% sodium azide). Cells were then incubated in cold buffer. Subsequent staining with monoclonal antibodies (mAbs) or isotype-matched controls was performed by incubation on ice for 30**min. Stained cells were then washed twice, resuspended in cold buffer, and analyzed using a FACSCalibur cell analyzer. More than 1×10^4^ cells were analyzed for each sample, and the results were processed using CellQuest software (BD, USA).

### Uptake of fluorescein isothiocyanate (FITC)-labeled dextran

Cultured DCs were washed twice and resuspended in 1**ml of RPMI-1640 supplemented with 10% FBS, 2**mM l-glutamine, 100**U/mL penicillin, 100**U/mL streptomycin, and 25**mM HEPES. The cells were then incubated with FITC-labeled dextran (0.2**mg/mL) at 4°C or 37°C for 1**h. Finally, the cells were washed twice and analyzed using a FACSCalibur cell analyzer.

### Autologous mixed leukocyte reaction (MLR)

PBMCs were obtained as described above, and naïve CD4^+^ T cells were purified from PBMCs using magnetic beads (Miltenyi Biotec). The autologous naïve CD4^+^ T cells obtained were resuspended at 1×10^5^ cells/well and incubated for 5 days in the presence of graded numbers of unstimulated DCs, LPS-treated DCs, or EV71 VLP-treated DCs. Incorporation of tritiated thymidine (1**µCi/well; New England Nuclear, Boston, MA, USA) for 16**h was determined using a liquid scintillation counter.

### Neutralization experiments

Human DCs were pre-incubated with 20**µg/mL neutralization Abs against TLR2 (clone TL2.1), TLR4 (clone HTA125), or IgG2a (isotype control) (eBioScience) for 1**h. EV71 VLPs were then added for 16**h. The cell culture supernatants were analyzed to determine the levels of IL-12 p70, IL-12 p40, and IL-10 by ELISA.

### Transfection of TLR4 in HEK293 cells

The pUNO-hTLR4, pDUO-MD2/TLR4, and pUNO empty plasmids were obtained from InvivoGen Inc. (San Diego, CA, USA). HEK293 cells (ATCC, Manassas, VA, USA) were plated in six-well tissue culture plates and maintained in Dulbecco’s minimum essential medium supplemented with 10% FBS. Cells were transfected using the Lipofectamine 2000 transfection protocol (Life Technologies Inc., Carlsbad, CA, USA) with 4.0**µg pUNO, pUNO-hTLR4, or pDUO-MD2/TLR4 and 6**µL Lipofectamine. Transfected cells were selected in medium containing 10**µg/mL blasticidin S-supplemented medium. Transfected cells were cultured in normal medium for 36–48**h and were then stimulated with medium alone, LPS (50**ng/mL), or EV71 VLPs (10**µg/mL) for 24**h. IL-8 protein in the culture supernatant was then measured by ELISA.

### Proteinase K treatment of EV71 VLPs

EV71 VLPs, LPS (positive control), or medium alone (negative control) were treated with proteinase K (1**µg/mL) at 37°C for 16**h prior to being added to DCs cultures. Then, EV71 VLPs, LPS, or medium alone treated with or without proteinase K were added to DCs for 24**h. The final concentration of proteinase K was 10**ng/mL, which did not affect the viability of DCs. Cell culture supernatants were analyzed to determine the levels of IL-12 p70, IL-12 p40, and IL-10 by ELISA.

### Western blotting

Total cell extracts were prepared using Gold lysis buffer (1% Triton X-100, 30**mM Tris pH**8.0, 137**mM sodium chloride, 15% glycerol, and 5**mM EDTA). Total protein (50**µg) was separated on 10% SDS-polyacrylamide minigels and transferred to Immobilon PVDF membranes (Millipore). The membranes were incubated overnight at 4°C with 10% bovine serum albumin in PBS to block binding of nonspecific immunoglobulins and then incubated with an anti-IκBα polyclonal Ab and an anti-α-tubulin mAb (both from Santa Cruz Biotechnology, Santa Cruz, CA, USA).

### Electrophoretic mobility shift assay (EMSA)

Nuclear extracts were prepared according to the manufacturer’s instructions (Pierce, USA). Nuclear extracts were subjected to EMSA as described previously [18].

### Statistical analysis

Statistical analysis was performed using one-way analysis of variance (ANOVA) followed by post-hoc Tukey analysis, and differences with *p* values of less than 0.05 were considered statistically significant.

## Results

### Binding of VLPs by DCs

Immature human DCs are highly specialized cells that are capable of efficient uptake and processing of Ags. Moreover, DCs can support the growth of several viruses, including EV71 [19]. However, the ability of DCs to bind EV71 has not been evaluated. In the present study, we exposed immature DCs to EV71 VLPs and used confocal microscopy and fluorescence-activated cell sorting (FACS) to monitor the association of EV71 VLPs with cell surfaces. Human DCs were incubated with EV71 VLPs for 30**min on ice to allow binding to the cell surface. We found that EV71 VLPs could bind to DCs in a concentration-dependent manner (Figure 1A and B). Incubation of DCs with 1**µg/mL EV71 VLPs for 30 or 120**min on ice indicated that binding to the surfaces of DCs was a time-dependent process, with FACS analysis indicating more extensive binding after 120**min than after 30**min (Figure 1C).

### Uptake of VLPs by DCs

The binding of particulate Ags to DCs may initiate signal transduction, resulting in cellular activation. To investigate this possibility, confocal microscopy and FACS analysis were used to study the interaction of human DCs with EV71 VLPs labeled with CFDA. We confirmed the uptake of EV71 VLPs by DCs (Figure 2A). Incubation of human DCs with various concentrations of CFDA-labeled EV71 VLPs for 120**min at 37°C showed that VLP uptake occurred in a concentration-dependent manner (Figure 2B). Moreover, incubation of human DCs with 10**µg/mL CFDA-labeled EV71 VLPs for 15, 30, or 120**min at 37°C showed that VLP uptake also occurred in a time-dependent manner (Figure 2C).

### VLPs induced production of cytokines and costimulatory molecules in human DCs

**Figure 3:**
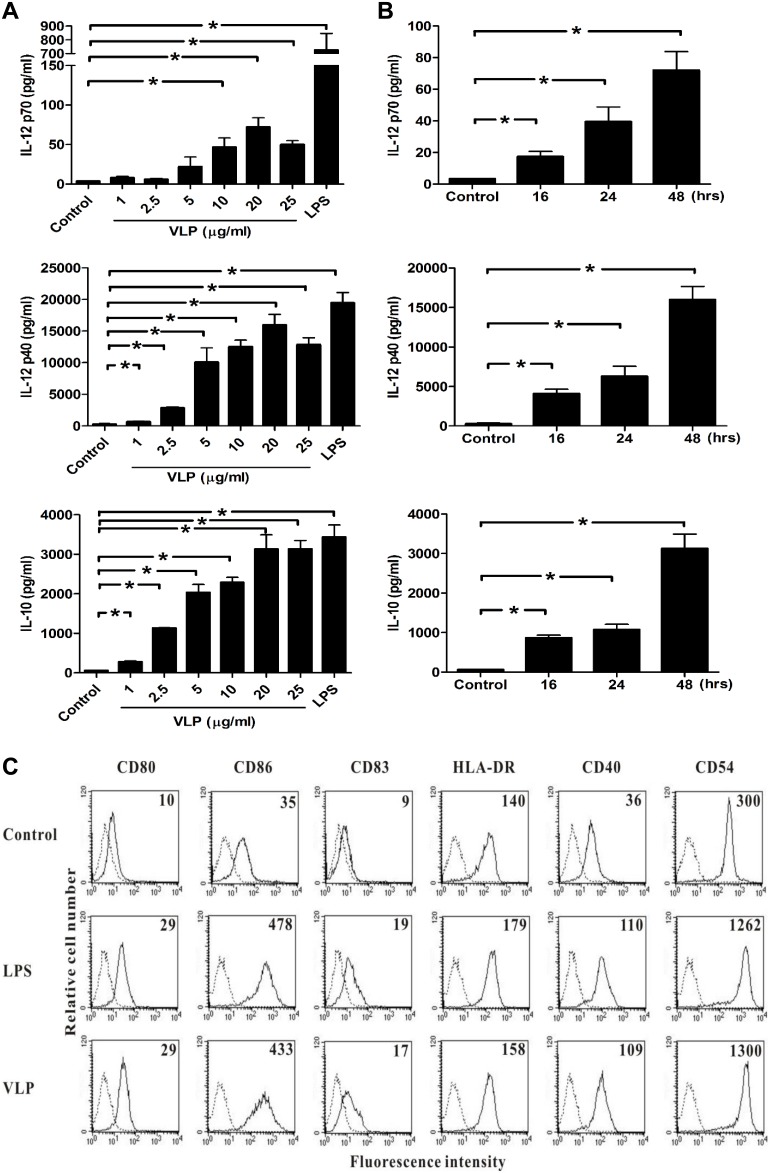
EV71 VLPs increased cytokines production and enhanced the abundance of cell-surface molecules in human DCs. (A) Human DCs were cultured for 24**h in the presence of 100**ng/mL LPS or various concentrations of EV71 VLPs. At the end of the incubation time, the culture medium was collected, and IL-12 p70, IL-12 p40, and IL-10 levels were measured using ELISA. (B) Human DCs were incubated with EV71 VLPs (10**µg/mL) for 16, 24, or 48**h. At the end of the incubation time, levels of secreted IL-12 p70, IL-12 p40, and IL-10 were analyzed by ELISA. (C) Human DCs were treated with EV71 VLPs (10**µg/mL; VLP), LPS (100**ng/mL; LPS), or medium alone (Control) for 24**h, and levels of surface markers were analyzed by flow cytometry (dotted line, isotype control; solid line, specific mAbs). The values shown in the flow cytometry profiles are the mean fluorescence intensity indices. Representative data from three different donors with similar results are shown. Values represent the means ± SEs of triplicate samples, and asterisks indicate statistically significant differences (*, *P*<0.05).

To determine whether EV71 VLPs affected the production of cytokines in human DCs, we compared cytokine concentrations in the supernatants of DCs cultured with different concentrations of EV71 VLPs. EV71 VLPs enhanced the production of IL-12 p70, IL-12 p40, and IL-10 (Figure 3A). Treatment of human DCs with EV71 VLPs (10**µg/mL) for 16, 24, or 48**h significantly enhanced the production of IL-12 p70, IL-12 p40, and IL-10 at all three time points (Figure 3B). It was clear that the stimulatory effects of EV71 VLPs on the production of IL-12 p70, IL-12 p40, and IL-10 were dependent on both concentration and time. Given its ability to induce DC activation and maturation [16], LPS was used as a positive control. To determine whether EV71 VLPs also modulated the maturation of human DCs *in vitro*, we compared the phenotypes of human DCs with or without a 24-h treatment with EV71 VLPs. EV71 VLPs showed increased expression of CD80, CD86, CD83, CD40, CD54, and HLA-DR on the cell membranes of human DCs (Figure 3C).

### VLPs reduced endocytic activity in human DCs

**Figure 4:**
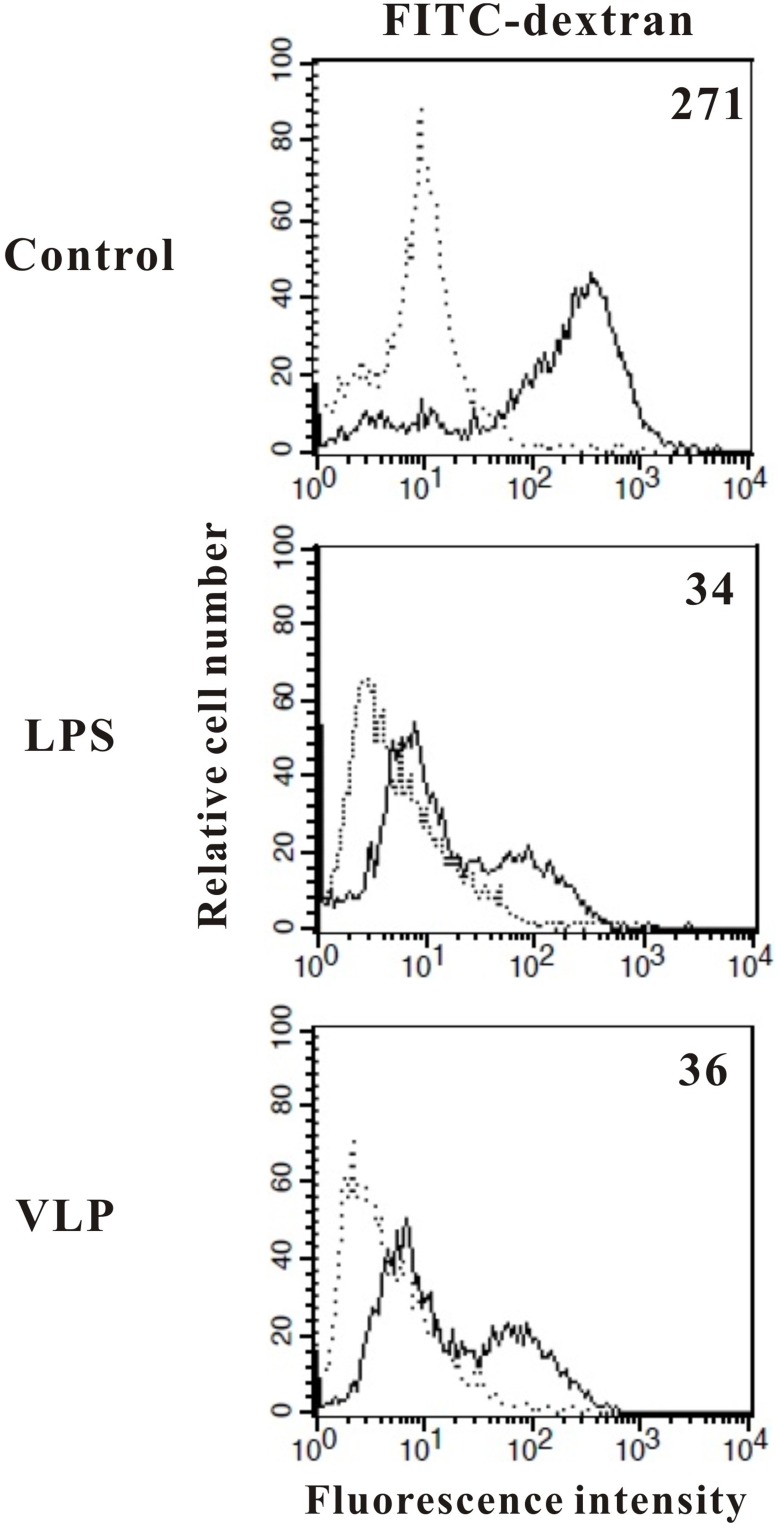
Effects of EV71 VLPs on the endocytic capacity of human monocyte-derived DCs. At day 5, immature DCs were stimulated with medium alone (Control), LPS (100**ng/mL; LPS), or EV71 VLPs (10**µg/mL; VLP) for 24**h and then incubated with FITC-dextran for 1**h at 4°C (dotted lines) or 37°C (solid lines). This experiment was repeated three times (from three different donors) with similar results. The values shown in the flow cytometry profiles are the mean fluorescence intensity indices.

The capacity of immature DCs to support high rates of endocytosis enables them to capture and process Ags with considerable efficiency. However, DCs lose their capacity to endocytose and process Ags as they mature and differentiate into potent immunostimulatory Ag-presenting cells [11]. Rates of uptake of FITC-dextran are higher in immature monocyte-derived DCs than in mature DCs; this uptake occurs through a combination of macropinocytosis and binding to the mannose receptor [20]. Given that LPS suppresses the endocytic capacity of DCs during their maturation [21], we evaluated whether EV71 VLPs affected the uptake of FITC-labeled dextran by human DCs. We showed that maturation of human DCs after exposure to EV71 VLPs was associated with reduced uptake of FITC-dextran (Figure 4), suggesting that EV71 VLPs suppressed endocytosis.

### VLPs enhanced the immunostimulatory capacity of DCs

**Figure 5:**
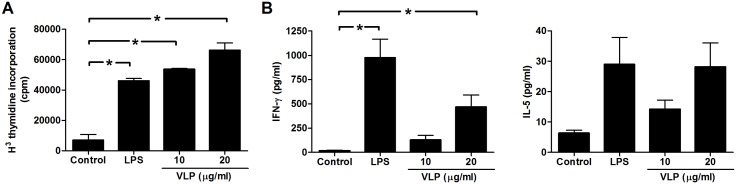
EV71 VLPs enhanced T-cell responses. (A) Immature DCs were stimulated with medium alone (Control), LPS (100**ng/mL), or EV71 VLPs (10 and 20**µg/mL; VLP) for 24**h. Proliferation of autologous T cells was measured after 5 days of coculture with DCs. (B) Supernatants were analyzed for IFN-γ and IL-5 produced by activated T cells after 2 days of culture. Representative data from three different donors with similar results are shown. Values represent the means ± SEs of triplicate samples, and asterisks indicate statistically significant differences (*, *P*<0.05).

Mature DCs induce much higher rates of proliferation in allogenic T cells than immature DCs [12]. Exposure of human DCs to EV71 VLPs increased the levels of cell-surface markers and IL-12 production. To test whether this maturation was sufficient to activate naive T cells, we compared the abilities of unstimulated immature DCs, LPS-treated DCs, and EV71 VLP-treated DCs to stimulate autologous T cells in an MLR. Immature DCs were first cultured in the presence or absence of EV71 VLPs or LPS for 48**h and were then cocultured with autologous T cells for 5 days. DCs that matured from immature DCs after treatment with either EV71 VLPs or LPS exhibited a higher immunostimulatory capacity than unstimulated immature DCs (Figure 5A). These results suggested that EV71 VLPs could induce the maturation and enhance the immunostimulatory capacity of DCs. In addition to determining the effects of EV71 VLPs on the immunostimulatory capacities of DCs, we also determined whether EV71 VLPs could stimulate cytokine secretion in the MLR system. After 48**h of MLR, the supernatants were examined to determine their expression of IFN-γ and IL-5. In an EV71 VLP- or LPS-treated DC/T-cell coculture system, IFN-γ expression was increased compared with that in the unstimulated DC/T-cell coculture system (Figure 5B). In contrast, stimulation of IL-5 production by EV71-VLPs or LPS was minimal and not significant (Figure 5B), and no effect of either treatment was observed on IL-4 levels (data not shown). These data provided strong evidence that EV71 VLPs affected T-cell-mediated responses by enhancing Th1-type cytokine expression.

### VLPs required TLR4 signaling to induce synthesis of IL-12 p40, IL-12 p70, and IL-10

**Figure 6:**
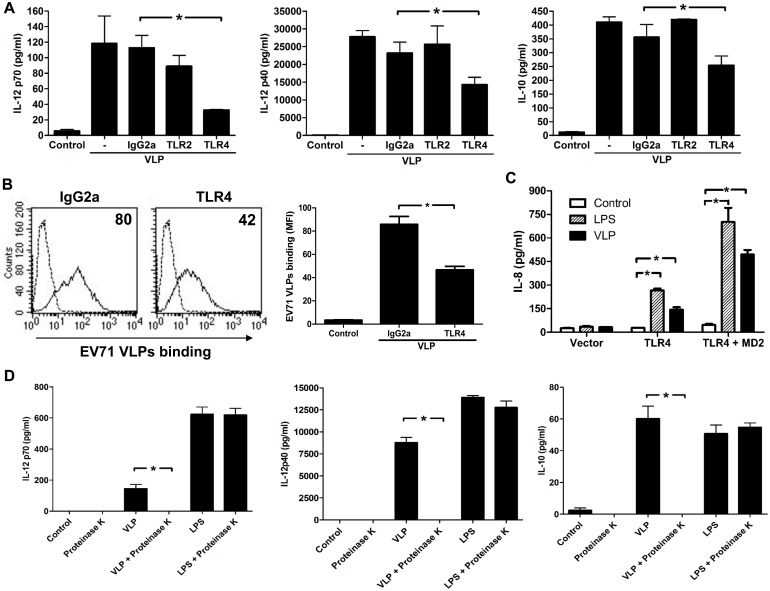
TLR4 mediated the maturation of DCs induced by EV71 VLPs. (A) Human DCs were pre-incubated with 20**µg/mL of anti-TLR2, anti-TLR4, or IgG2a (as an isotype control) separately for 1**h and were then challenged with EV71 VLPs (10**µg/mL) for 16**h. The control was not treated with EV71 VLPs. Supernatants from the cell cultures were collected to determine levels of IL-12 p70, IL-12 p40, and IL-10. Significant differences in the levels of cytokines produced by DCs treated with Abs and those treated with IgG2a are indicated by asterisks (*p<0.05). (B) Human DCs were pre-incubated with 20**µg/mL of anti-TLR4 mAb or IgG2a (as an isotype control) separately for 1**h and were then treated with 10**µg/mL EV71 VLPs for 30**min on ice to allow binding to the cell surface. Surface-bound EV71 VLPs were measured with flow cytometry through immunostaining with anti-EV71 monoclonal antibodies. The control cells (no VLPs; dotted line) were stained with the same primary antibody followed by a secondary antibody. The bar diagram shows the MFI with statistics for the anti-TLR4 mAb effect on binding of EV71 VLPs to DCs. (C) HEK293 cells were transfected with control plasmids, TLR4, or TLR4+MD2. The transfected cells were stimulated with medium alone, LPS (50**ng/mL), or EV71 VLP (10**µg/mL) for 24**h, and IL-8 protein in the supernatants was measured by ELISA. This experiment was repeated three times with similar results. (D) Proteinase K-treated EV71 VLPs abrogated the production of IL-12 p70, IL-12 p40, and IL-10 by DCs. Human DCs were incubated with medium alone (Control), proteinase K (10**ng/mL), EV71 VLPs (10**µg/mL), proteinase K-treated EV71 VLPs, LPS (100**ng/mL), or proteinase K-treated LPS for 24**h. At the end of the incubation time, the production of IL-12 p70, IL-12 p40, and IL-10 was analyzed by ELISA. Representative data from three different donors with similar results are shown. Values represent the means ± SEs of triplicate samples, and asterisks indicate statistically significant differences (*, *P*<0.05).

TLRs contribute to the antifungal defense mechanism in *Drosophila* and to antibacterial defense mechanisms in humans. Neutralization experiments were performed to examine the role of these receptors in the signal transduction triggered by EV71 VLPs in DCs. Neutralizing Abs were used to block the TLR2 and TLR4 receptors on the surface of DCs before they were treated with EV71 VLPs. The addition of an anti-TLR4 mAb to DCs significantly decreased the ability of EV71 VLPs (10**µg/mL) to enhance IL-12 p40, IL-12 p70, and IL-10 production by approximately 48%, 68%, and 41%, respectively, whereas the addition of an anti-TLR2 mAb failed to inhibit the increased production of IL-12 p40, IL-12 p70, and IL-10 after exposure to EV71 VLPs (Figure 6A). We also used an anti-TLR4 neutralized mAb to perform EV71 VLP binding assays in DCs. We found that anti-TLR4 mAbs partially inhibited EV71 VLP binding to DCs (Figure 6B). To further confirm that EV71 VLPs utilized TLR4, we tested whether EV71 VLPs could active HEK293 cells expressing functional TLR4 by assessing IL-8 production. The upregulation of *IL-8* gene transcription is known to involve the activation of the NF-κB signaling pathway. Thus, we next analyzed the capacity of EV71 VLPs or LPS to induce IL-8 production by transfected HEK293 cells (Figure 6C). TLR4- or TLR4/MD2-transfected HEK293 cells produced IL-8 in response to EV71 VLPs or LPS. However, medium alone did not induce significant IL-8 production from HEK293 cells transfected with TLR4 or TLR4/MD2. Thus, these results suggested that TLR4 was involved in EV71 VLP binding and regulated IL-12 p40, IL-12 p70, and IL-10 expression in DCs. To rule out the possibility of endotoxin contamination in EV71 VLPs, we used proteinase K to degrade the EV71 VLPs because proteinase K exhibits broad substrate specificity and degrades many proteins [22]. The results clearly indicated that while EV71 VLPs effectively induced the production of IL-12 p70, IL-12 p40, and IL-10 in DCs, prior treatment of EV71 VLPs with proteinase K blocked their ability to induce the production of IL-12 p70, IL-12 p40, or IL-10 (Figure 6D). Treatment of LPS (positive control) with proteinase K failed to affect the ability of LPS to induce the production of IL-12 p70, IL-12 p40, and IL-10 by DCs. This result indicated that the EV71 VLPs were not contaminated with endotoxin and suggested that TLR4 was required for EV71 VLP-mediated stimulation of the expression of IL-12 p40, IL-12 p70, and IL-10.

### VLPs induced IκBα degradation and NF-κB activation in DCs

**Figure 7:**
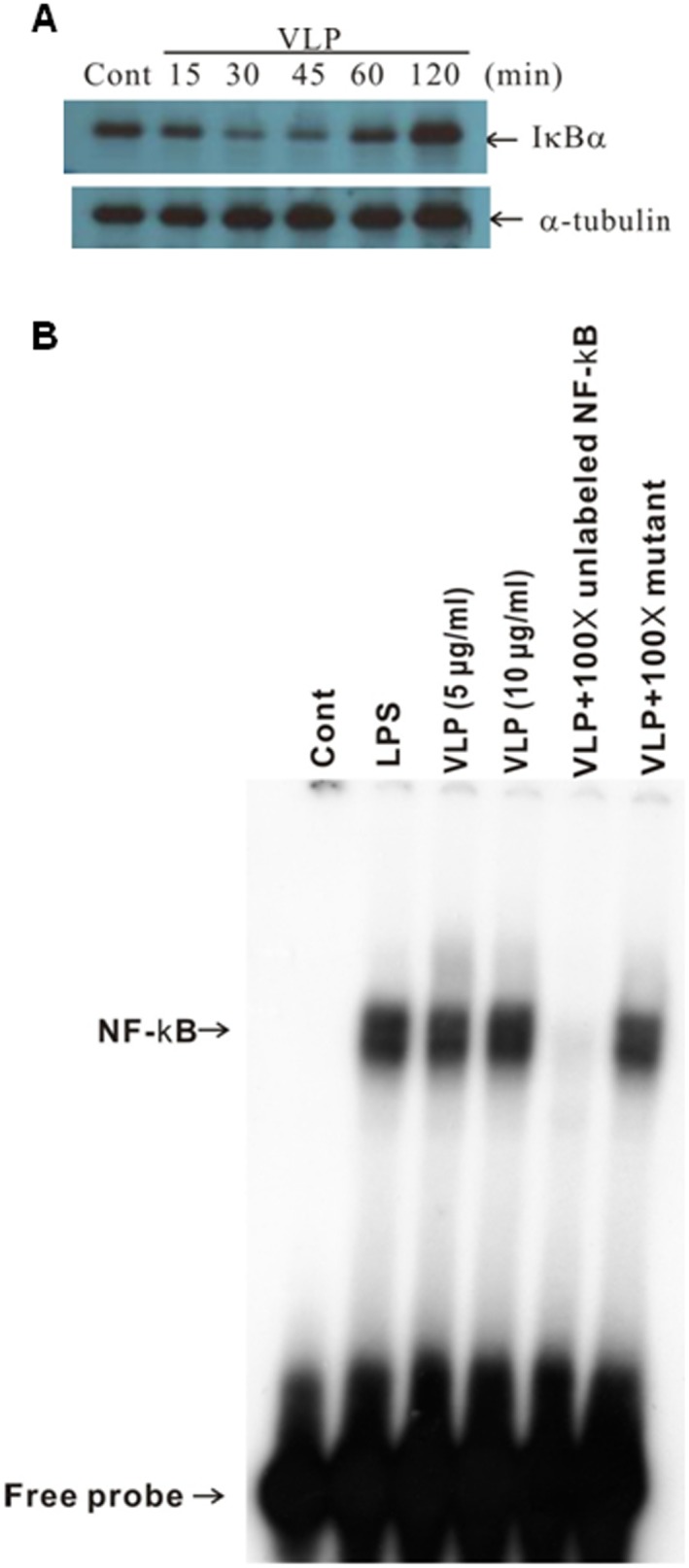
Induction of IκBα degradation and NF-κB activation by incubation of human DCs with EV71 VLPs. (A) Human DCs were treated with EV71 VLPs (10**µg/mL) for the indicated times. Western blotting was used to analyze cytosolic fractions for degradation of IκBα. The lower panel shows anti-α-tubulin staining of the same blot and confirmed equal loading of all samples. (B) Human monocyte-derived DCs were not treated (Cont) or were treated with LPS (100**ng/mL) or EV71 VLPs (5 or 10**µg/mL) for 2**h, and nuclear fractions were prepared. An electrophoretic mobility shift assay was used to assay NF-κB binding activity in the nuclear fractions. To assess the specificity of the binding, a 100-fold excess of cold NF-κB probe (a double-stranded NF-κB oligonucleotide, 5′-AGTTGAGG**G**GACTTTCCCAGGC-3′) or mutant probe (a double-stranded mutated oligonucleotide, 5′-AGTTGA**GGCGACTTTCCC**AGGC-3′) was added to the EV71 VLP condition. A double-stranded mutated probe, 5′-AGTTGAGG**C**GACTTTCCCAGGC-3′, was used to examine the specificity of binding of NF-κB to DNA (the underlined sequence is identical to the κB consensus sequence except for a **G**-to-**C** substitution in the NF-κB DNA binding motif). This experiment was repeated three times (from three different donors) with similar results.

Members of the NF-κB family of transcription factors are activated in the course of DC maturation [23]. NF-κB normally binds to IκBα, which blocks translocation of NF-κB from the cytoplasm to the nucleus. Phosphorylation of IκBα upon exposure of cells to inflammatory stimuli, including LPS and TNF-α, triggers the degradation of IκBα and nuclear translocation of NF-κB. Western blotting, which was used to examine whether EV71 VLPs affected IκBα degradation, indicated significant degradation of cytosolic IκBα within 30**min after activation of DCs with EV71 VLPs (Figure 7A). To determine whether EV71 VLPs activated NF-κB, DCs were cultured in the presence of EV71 VLPs for 2**h, and EMSA was performed to determine NF-κB binding to DNA. EV71 VLPs could induce NF-κB translocation and activation (Figure 7B). Identical results were obtained after treating DCs with LPS. The binding of NF-κB was specific and was blocked by unlabeled NF-κB-specific competitor oligonucleotides.

## Discussion

Immunization with VLPs, which are empty particles that comprise viral capsid proteins alone, has been shown to protect against various infectious diseases [7]. Given that immunogens containing viral nucleic acids may cause undesirable side effects [24], VLPs offer a safer alternative to intact viruses for clinic trials and routine vaccination programs [25], [26]. Studies of VLPs derived from important viruses, such as human immunodeficiency virus and Norwalk virus, have recently indicated that VLPs can induce the production of neutralizing Abs and cytotoxic T-lymphocyte responses [27]. Moreover, VLPs of hepatitis B virus (e.g., Recombivax HB, Merck) and human papillomavirus (Gardisil, Merck) have been licensed for use as vaccines [28]. In 2008, we found that EV71 VLPs prevent EV71 infection by increasing the titers of neutralizing Abs in mice [29]. Recently, we also found that monkeys intramuscularly immunized with EV71 VLPs showed potent humoral and cellular immune responses to both EV71 VLPs and EV71 virions upon in**vitro stimulation [17]. Therefore, EV71 VLPs are a candidate vaccine for the prevention of EV71 infection. However, whether the immunized monkeys are protected from future infection remains to be investigated. Given that DCs are required for the initiation of a potent cellular immune response, the immunogenicity of vaccines may be dependent on the DC response. In this study, we investigated the interactions of EV71 VLPs with human DCs. We showed that EV71 VLPs bound to and activated DCs and identified TLR4 as a receptor for EV71 VLPs. Moreover, pulsing of human DCs with EV71 VLPs induced T-cell responses that were characterized by IFN-γ secretion. This finding is similar to the results of our monkey study, in which the PBMCs from monkeys immunized with the EV71 VLP vaccine were stimulated with EV71 VLPs or inactivated EV71 as antigens; both groups of monkeys were able to induce T cell proliferation and higher levels of IFN-γ secretion. Therefore, based on our current findings in human DCs *in vitro*, we speculated that DCs play an important role in presenting EV71 VLPs antigen to T cells, and may generate a memory immune response to EV71 in EV71 VLPs immunized monkeys.

IL-12 p40 partners with the p35 and p19 polypeptides to generate the heterodimeric cytokines IL-12 and IL-23, respectively. These cytokines play critical and distinct roles in host defense. During immune responses, IL-12 plays a central role as a link between the innate and adaptive immune systems. Secretion of IL-12 is a critical component of the immune regulation of B and T lymphocytes after exposure to an infectious agent [30]. IL-12 induces and promotes T cells and natural killer cells to generate IFN-γ and lyse infected cells. IL-12 also polarizes the immune system towards a primary Th-1 response. Thus, our data strongly suggested that DCs activated by EV71 VLPs may be highly effective inducers of Ag-specific cellular immunity. We found that immature DCs pulsed with EV71 VLPs induced the proliferation of syngeneic T cells, which initiated a strongly polarized Th1 immune response through production of IFN-γ. Given that DCs are critically involved in the differentiation and isotype switching of B cells [31], VLP-activated DCs may also facilitate the substantial Ab production observed after vaccination with VLPs in the absence of an adjuvant.

IL-23 is mainly secreted by activated macrophages and DCs. IL-23 is not as efficient as IL-12 in the induction of IFN-γ production and in the polarization of T cells to the Th1 pattern. On the other hand, IL-23 is more effective than IL-12 in the induction of memory T-cell proliferation. However, in addition to its role in memory T-cell responses, IL-23 drives the development of a novel T-cell subset characterized by the production of IL-17 (Th17), which plays a central role in mediating many chronic inflammatory responses. In this study, we found that EV71 VLPs could not induce IL-23 production in human DCs and could not stimulate Th17 cell production in T cell cultures of MLR. Although we detected no IL-23 or IL-17 production, Chen et al. recently demonstrated that the frequencies of Th17 cells and the number of neutrophils in peripheral blood samples from children infected with EV71 were significantly higher compared to controls [32]. In addition, higher levels of IL-17 and IL-23 were found in sera, but lower IFN-γ production was observed during EV71 infections. We speculate that DCs infected with EV71 may induce IL-23 production and enhance the expansion of Th17 cells. Further studies are required to elucidate these mechanisms.

Type I IFNs are potent antiviral cytokines that influence DC maturation and many other immune processes. In this study, we detect no induction of interferon-α in human DCs treated with EV71 VLPs. Recently, we detected high levels of interferon-α production in plasmacytoid DCs (pDCs) incubated with EV71, but not with EV71 VLPs (unpublished data). It is possible that the genomic RNA remaining in the EV71 triggers various nucleic acid sensors, such as TLR7, in pDCs and mediates additional immune activation with strong type I IFN production.

DCs are central players of the immune response. Two major types of naturally occurring DCs circulate in peripheral blood, myeloid DCs (mDCs) and pDCs. These different DCs express different surface molecules and have distinct functions. mDCs and pDCs express different but complementary TLRs, which allows them to respond to different types of pathogens. mDCs recognize diverse pathogens due to their broad TLR expression and produce IL-12 after activation. pDCs specifically recognize pathogens containing single-stranded RNA (ssRNA) via TLR7 and unmethylated CpG DNA motifs via TLR9; additionally, pDCs produce higher levels of IFN-α than other types of blood cells in response to viruses [33], [34]. Schulte et al. demonstrated that primary human myeloid BDCA1^+^ DCs infected with different enteroviruses have differential susceptibilities and responses [35]. Thus, the effect of EV71 VLPs on naturally occurring mDCs and pDCs may differ somewhat from monocyte-derived DCs. This requires additional studies to clarify.

Many studies have focused on the application of human papillomavirus-like particles to DCs. The acute activation of DCs by human papillomavirus-like particles [36] requires signaling events that involve TLR4 and NF-κB [37]. The uptake of human papillomavirus VLPs by DCs is mediated by Fcγ receptors and contributes to the acquisition of T-cell immunity [38]. These findings explain the striking ability of vaccines based on papillomavirus VLPs to induce potent T- and B-cell responses.

Pattern recognition receptors (PRRs) of the innate immune system have recently been shown to recognize conserved structural features of microbial Ags [39]. Given that DCs express many types of PRRs, including the mannose receptor DEC205 and multiple TLRs, PRRs may be important components of the innate immune system involved in the initiation of Ag-specific immune responses. However, pattern recognition of the structural elements of virions by DCs is poorly understood. Confocal microscopy and flow cytometry enabled us to use binding and uptake experiments to characterize receptor-ligand interactions early after immature DCs encountered EV71 VLPs. We found that EV71 VLPs could bind to and be taken up by human DCs. Neutralization experiments with Abs that block TLR2 and TLR4 further indicated that TLR4 plays a critical role in the signal transduction cascade induced by EV71 VLPs. We also used TLR4- and TLR4/MD2-transfected HEK293 cells that produced IL-8 in response to EV71 VLPs to confirm these results. Our data demonstrated that TLR4 was required for LPS or EV71 VLPs to active IL-8 generation. When MD2, a protein associated with the extracellular domain of TLR4, was transfected with TLR4 in HEK293 cells, MD2 enhanced TLR4-mediated production of IL-8 by LPS or EV71 VLPs. Although many studies have reported that MD2 and TLR4 are required for LPS-induced responses, Dueñas et al. observed that HEK293 cells transfected with TLR4 alone could induce the kB-driven transcriptional activity in response to *Escherichia coli* LPS. However, cotransfection of TLR4 and MD2 provided optimal induction. The response to *Brucella* spp. and *Ochrobactrum anthropi* LPS was only significant at high concentrations. Thus, their data indicated that LPS from *Brucella* spp. and *O. anthropic*, which contain lipid A moieties with structural features different from those of *Enterobacteriaceae*, elicit biochemical signaling via TLR4 only at high concentrations [40]. Yang et al. also found that HEK293 cells transfected with TLR4 alone can stimulate NF-κB reporter activity and IL-8 production but not Elk-1 activity in response to *E. coli* LPS [41]. In our current study, our LPS was from *E. coli*; this may explain why only TLR4-transfected HEK293 cells produced IL-8 after LPS treatment. From our results, we confirmed that EV71 VLPs engaged TLR4 molecules on the surfaces of DCs. Moreover, MD2 enhanced TLR4-mediated production of IL-8 by EV71 VLPs or LPS in HEK293 cells. MD2 has a unique hydrophobic cavity that directly binds to lipid A, the active center of LPS [42], [43]. However, the mechanism linking MD2 to EV71 VLP responses remains unknown. We speculate that EV71 VLPs may interact with a large hydrophobic pocked in MD2. Future crystallographic studies of the structure of EV71 VLPs with TLR4-MD2 may provide critical clues to these mechanisms. In neutralization experiments, Abs that block TLR4 cannot completely inhibit EV71 VLP binding with DCs. Therefore, we cannot exclude the possibility that EV71 VLPs can bind with other receptors. Li et al. found that EV71 infection of immature human DCs prior to their expression of viral Ag is partially mediated by the C-type lectin receptor DC-SIGN [19]. Recently, Ren et al. also demonstrated that DC-SIGN captures EV71 and promotes monocyte-derived DC-mediated viral transinfection [44]. Human P-selectin glycoprotein ligand-1 (PSGL-1) [45] and human scavenger receptor class B, member 2 (hSCARB2) [46], [47] have been identified as cellular receptors for EV71 [48]. Several EV71 isolates bind PSGL-1 to infect lymphocyte cell lines. Other EV71 isolates and CVA16 infect lymphocytes independently of PSGL-1; these viruses may interact with SCARB2 or with another unidentified receptor. SCARB2 is present on epithelial cells and fibroblasts, which do not express PSGL-1, but can be infected by several EV71 isolates and by CVA16, which depends on SCARB2. To date, no studies have investigated EV71 with PSGL-1 or SCARB2 in DCs. Nevertheless, the details of PSGL-1- or SCARB2-mediated entry for EV71 VLPs or EV71 in monocyte-derived DCs, mDCs, or pDCs still needs more exploration.

Regarding the signal transduction pathways involved in the maturation of human DCs by EV71 VLPs, our demonstration that the NF-κB pathway was activated when immature DCs were exposed to EV71 VLPs suggested that this pathway played a role in the maturation process. The presence of NF-κB-response elements in the promoters of many of the genes that encode pro-inflammatory cytokines suggests a major role of the NF-κB pathway in driving immune responses [23]. For instance, the promoters of the genes that encode hIL-12 p35 and hIL-12 p40 contain binding sites for NF-κB [49]. It is likely that NF-κB is also involved in the expression of genes that encode IL-12 p35 and IL-12 p40.

**Figure 8:**
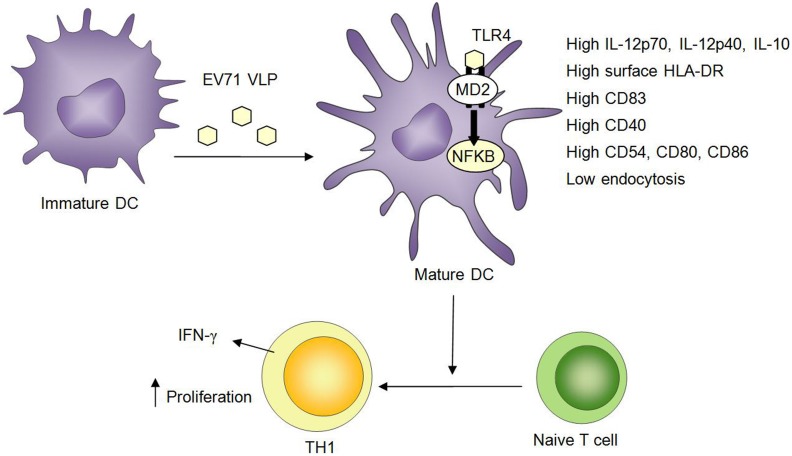
The maturation and activation of DCs induced by EV71 VLPs required TLR4 and NF-κB activity and enabled naïve T cells to initiate Th1-cell responses.

In conclusion, the results of the current study revealed that TLR4 contributed to the targeting of EV71 VLPs to DCs. Additionally, our study provided insights into the mechanisms through which the maturation of DCs induced by EV71 VLPs facilitates the presentation of peptides derived from EV71 VLPs to naïve Ag-specific T cells (Figure 8). The ability of EV71 VLPs to trigger the activation and maturation of DCs through signaling that involves TLR4 and NF-κB may account for the high intrinsic immunogenicity of EV71 VLPs.

## References

[1] Tagaya I, Takayama R, Hagiwara A (1981) A large-scale epidemic of hand, foot and mouth disease associated with enterovirus 71 infection in Japan in 1978. Jpn J Med Sci Biol 34: 191–196. 6273621 10.7883/yoken1952.34.191

[2] Wang SM, Lei HY, Yu CK, Wang JR, Su IJ, et al (2008) Acute chemokine response in the blood and cerebrospinal fluid of children with enterovirus 71-associated brainstem encephalitis. J Infect Dis 198: 1002–1006. 18710325 10.1086/591462

[3] Wu WH, Kuo TC, Lin YT, Huang SW, Liu HF, et al (2013) Molecular epidemiology of enterovirus 71 infection in the central region of Taiwan from 2002 to 2012. PLoS One 8: e83711. 24391812 10.1371/journal.pone.0083711 PMC3877097

[4] Wu CN, Lin YC, Fann C, Liao NS, Shih SR, et al (2001) Protection against lethal enterovirus 71 infection in newborn mice by passive immunization with subunit VP1 vaccines and inactivated virus. Vaccine 20: 895–904. 11738755 10.1016/s0264-410x(01)00385-1

[5] McMinn PC (2002) An overview of the evolution of enterovirus 71 and its clinical and public health significance. FEMS Microbiol Rev 26: 91–107. 12007645 10.1111/j.1574-6976.2002.tb00601.x

[6] Zeltins A (2013) Construction and characterization of virus-like particles: a review. Mol Biotechnol 53: 92–107. 23001867 10.1007/s12033-012-9598-4 PMC7090963

[7] Vacher G, Kaeser MD, Moser C, Gurny R, Borchard G (2013) Recent advances in mucosal immunization using virus-like particles. Mol Pharm 10: 1596–1609. 23548071 10.1021/mp300597g

[8] Grgacic EV, Anderson DA (2006) Virus-like particles: passport to immune recognition. Methods 40: 60–65. 16997714 10.1016/j.ymeth.2006.07.018 PMC7128828

[9] Hu YC, Hsu JT, Huang JH, Ho MS, Ho YC (2003) Formation of enterovirus-like particle aggregates by recombinant baculoviruses co-expressing P1 and 3CD in insect cells. Biotechnol Lett 25: 919–925. 12889824 10.1023/a:1024071514438

[10] Chung YC, Huang JH, Lai CW, Sheng HC, Shih SR, et al (2006) Expression, purification and characterization of enterovirus-71 virus-like particles. World J Gastroenterol 12: 921–927. 16521221 10.3748/wjg.v12.i6.921 PMC4066158

[11] Banchereau J, Steinman RM (1998) Dendritic cells and the control of immunity. Nature 392: 245–252. 9521319 10.1038/32588

[12] Cella M, Sallusto F, Lanzavecchia A (1997) Origin, maturation and antigen presenting function of dendritic cells. Curr Opin Immunol 9: 10–16. 9039784 10.1016/s0952-7915(97)80153-7

[13] Aerts-Toegaert C, Heirman C, Tuyaerts S, Corthals J, Aerts JL, et al (2007) CD83 expression on dendritic cells and T cells: correlation with effective immune responses. Eur J Immunol 37: 686–695. 17301951 10.1002/eji.200636535

[14] Krieg AM (2002) CpG motifs in bacterial DNA and their immune effects. Annu Rev Immunol 20: 709–760. 11861616 10.1146/annurev.immunol.20.100301.064842

[15] Korokhov N, Noureddini SC, Curiel DT, Santegoets SJ, Scheper RJ, et al (2005) A single-component CD40-targeted adenovirus vector displays highly efficient transduction and activation of dendritic cells in a human skin substrate system. Mol Pharm 2: 218–223. 15934782 10.1021/mp050002w

[16] Ausiello CM, Fedele G, Urbani F, Lande R, Di Carlo B, et al (2002) Native and genetically inactivated pertussis toxins induce human dendritic cell maturation and synergize with lipopolysaccharide in promoting T helper type 1 responses. J Infect Dis 186: 351–360. 12134231 10.1086/341510

[17] Lin YL, Yu CI, Hu YC, Tsai TJ, Kuo YC, et al (2012) Enterovirus type 71 neutralizing antibodies in the serum of macaque monkeys immunized with EV71 virus-like particles. Vaccine 30: 1305–1312. 22214888 10.1016/j.vaccine.2011.12.081

[18] Lin YL, Liang YC, Tseng YS, Huang HY, Chou SY, et al (2009) An immunomodulatory protein, Ling Zhi-8, induced activation and maturation of human monocyte-derived dendritic cells by the NF-kappaB and MAPK pathways. J Leukoc Biol 86: 877–889. 19498044 10.1189/jlb.0708441

[19] Lin YW, Wang SW, Tung YY, Chen SH (2009) Enterovirus 71 infection of human dendritic cells. Exp Biol Med (Maywood) 234: 1166–1173. 19596831 10.3181/0903-RM-116

[20] Kato M, Neil TK, Fearnley DB, McLellan AD, Vuckovic S, et al (2000) Expression of multilectin receptors and comparative FITC-dextran uptake by human dendritic cells. Int Immunol 12: 1511–1519. 11058570 10.1093/intimm/12.11.1511

[21] Chou NT, Cheng CF, Wu HC, Lai CP, Lin LT, et al.. (2012) Chlorella sorokiniana-Induced Activation and Maturation of Human Monocyte-Derived Dendritic Cells through NF-kappa B and PI3K/MAPK Pathways. Evidence-Based Complementary and Alternative Medicine.

[22] Petsch D, Deckwer WD, Anspach FB (1998) Proteinase K digestion of proteins improves detection of bacterial endotoxins by the Limulus amebocyte lysate assay: application for endotoxin removal from cationic proteins. Anal Biochem 259: 42–47. 9606141 10.1006/abio.1998.2655

[23] Ghosh S, May MJ, Kopp EB (1998) NF-kappa B and Rel proteins: evolutionarily conserved mediators of immune responses. Annu Rev Immunol 16: 225–260. 9597130 10.1146/annurev.immunol.16.1.225

[24] Ramqvist T, Andreasson K, Dalianis T (2007) Vaccination, immune and gene therapy based on virus-like particles against viral infections and cancer. Expert Opin Biol Ther 7: 997–1007. 17665989 10.1517/14712598.7.7.997

[25] Szarewski A (2010) HPV vaccine: Cervarix. Expert Opin Biol Ther 10: 477–487. 20132062 10.1517/14712591003601944

[26] Buonaguro FM, Tornesello ML, Buonaguro L (2009) Virus-like particle vaccines and adjuvants: the HPV paradigm. Expert Rev Vaccines 8: 1379–1398. 19803760 10.1586/erv.09.81

[27] Young KR, McBurney SP, Karkhanis LU, Ross TM (2006) Virus-like particles: designing an effective AIDS vaccine. Methods 40: 98–117. 16997718 10.1016/j.ymeth.2006.05.024

[28] Shi L, Sings HL, Bryan JT, Wang B, Wang Y, et al (2007) GARDASIL: prophylactic human papillomavirus vaccine development–from bench top to bed-side. Clin Pharmacol Ther 81: 259–264. 17259949 10.1038/sj.clpt.6100055

[29] Chung YC, Ho MS, Wu JC, Chen WJ, Huang JH, et al (2008) Immunization with virus-like particles of enterovirus 71 elicits potent immune responses and protects mice against lethal challenge. Vaccine 26: 1855–1862. 18329759 10.1016/j.vaccine.2008.01.058

[30] Trinchieri G (1993) Interleukin-12 and its role in the generation of TH1 cells. Immunol Today 14: 335–338. 8103338 10.1016/0167-5699(93)90230-I

[31] Fayette J, Durand I, Bridon JM, Arpin C, Dubois B, et al (1998) Dendritic cells enhance the differentiation of naive B cells into plasma cells in**vitro. Scand J Immunol 48: 563–570. 9874489 10.1046/j.1365-3083.1998.00471.x

[32] Chen JG, Tong J, Liu HB, Liu YZ, Su ZL, et al (2012) Increased frequency of Th17 cells in the peripheral blood of children infected with enterovirus 71. Journal of Medical Virology 84: 763–767. 22431024 10.1002/jmv.23254

[33] MacDonald KPA, Munster DJ, Clark GJ, Dzionek A, Schmitz J, et al (2002) Characterization of human blood dendritic cell subsets. Blood 100: 4512–4520. 12393628 10.1182/blood-2001-11-0097

[34] Schreibelt G, Tel J, Sliepen KHEWJ, Benitez-Ribas D, Figdor CG, et al (2010) Toll-like receptor expression and function in human dendritic cell subsets: implications for dendritic cell-based anti-cancer immunotherapy. Cancer Immunology Immunotherapy 59: 1573–1582. 20204387 10.1007/s00262-010-0833-1 PMC11029854

[35] Schulte BM, Kers-Rebel ED, Prosser AC, Galama JMD, van Kuppeveld FJM, et al.. (2013) Differential Susceptibility and Response of Primary Human Myeloid BDCA1(+) Dendritic Cells to Infection with Different Enteroviruses. Plos One 8. 10.1371/journal.pone.0062502 PMC3634769 23638101

[36] Lenz P, Day PM, Pang YY, Frye SA, Jensen PN, et al (2001) Papillomavirus-like particles induce acute activation of dendritic cells. J Immunol 166: 5346–5355. 11313370 10.4049/jimmunol.166.9.5346

[37] Yan M, Peng J, Jabbar IA, Liu X, Filgueira L, et al (2005) Activation of dendritic cells by human papillomavirus-like particles through TLR4 and NF-kappaB-mediated signalling, moderated by TGF-beta. Immunol Cell Biol 83: 83–91. 15661045 10.1111/j.1440-1711.2004.01291.x

[38] Da Silva DM, Fausch SC, Verbeek JS, Kast WM (2007) Uptake of human papillomavirus virus-like particles by dendritic cells is mediated by Fcgamma receptors and contributes to acquisition of T cell immunity. J Immunol 178: 7587–7597. 17548594 10.4049/jimmunol.178.12.7587

[39] Coll RC, O'Neill LA (2010) New insights into the regulation of signalling by toll-like receptors and nod-like receptors. J Innate Immun 2: 406–421. 20505309 10.1159/000315469

[40] Duenas AI, Orduna A, Crespo MS, Garcia-Rodriguez C (2004) Interaction of endotoxins with Toll-like receptor 4 correlates with their endotoxic potential and may explain the proinflammatory effect of Brucella spp. LPS. Int Immunol 16: 1467–1475. 15339879 10.1093/intimm/dxh148

[41] Yang H, Young DW, Gusovsky F, Chow JC (2000) Cellular events mediated by lipopolysaccharide-stimulated toll-like receptor 4. MD-2 is required for activation of mitogen-activated protein kinases and Elk-1. J Biol Chem 275: 20861–20866. 10877845 10.1074/jbc.M002896200

[42] Park BS, Song DH, Kim HM, Choi BS, Lee H, et al (2009) The structural basis of lipopolysaccharide recognition by the TLR4-MD-2 complex. Nature 458: 1191–1195. 19252480 10.1038/nature07830

[43] Plevka P, Perera R, Cardosa J, Kuhn RJ, Rossmann MG (2012) Crystal structure of human enterovirus 71. Science 336: 1274. 22383808 10.1126/science.1218713 PMC3448362

[44] Ren XX, Ma L, Liu QW, Li C, Huang Z, et al.. (2014) The molecule of DC-SIGN captures enterovirus 71 and confers dendritic cell-mediated viral trans-infection. Virology Journal 11. 10.1186/1743-422X-11-47 PMC3995660 24620896

[45] Nishimura Y, Shimojima M, Tano Y, Miyamura T, Wakita T, et al (2009) Human P-selectin glycoprotein ligand-1 is a functional receptor for enterovirus 71. Nat Med 15: 794–797. 19543284 10.1038/nm.1961

[46] Yamayoshi S, Yamashita Y, Li J, Hanagata N, Minowa T, et al (2009) Scavenger receptor B2 is a cellular receptor for enterovirus 71. Nat Med 15: 798–801. 19543282 10.1038/nm.1992

[47] Yamayoshi S, Koike S (2011) Identification of a human SCARB2 region that is important for enterovirus 71 binding and infection. J Virol 85: 4937–4946. 21389126 10.1128/JVI.02358-10 PMC3126200

[48] Yamayoshi S, Ohka S, Fujii K, Koike S (2013) Functional Comparison of SCARB2 and PSGL1 as Receptors for Enterovirus 71. Journal of Virology 87: 3335–3347. 23302872 10.1128/JVI.02070-12 PMC3592140

[49] Yoshimoto T, Kojima K, Funakoshi T, Endo Y, Fujita T, et al (1996) Molecular cloning and characterization of murine IL-12 genes. J Immunol 156: 1082–1088. 8557982

